# Supplementary material for: DMRforPairs: identifying Differentially Methylated Regions between unique samples using array based methylation profiles
Source: BMC Bioinformatics. 2014 May 15;15:141. doi: 10.1186/1471-2105-15-141 (PMC4046028; doi:10.1186/1471-2105-15-141)
Supplement: Additional file 2 — DMRforPairs output for the comparison of A431-MCF7 and NA17018-NA17105. Please start from the HTML files in each folder. Available via the BMC Bioinformatics website. [file 1471-2105-15-141-S2.zip › 1394847754114233_MOESM2_ESM/A431_MCF7/figures/5375.pdf]

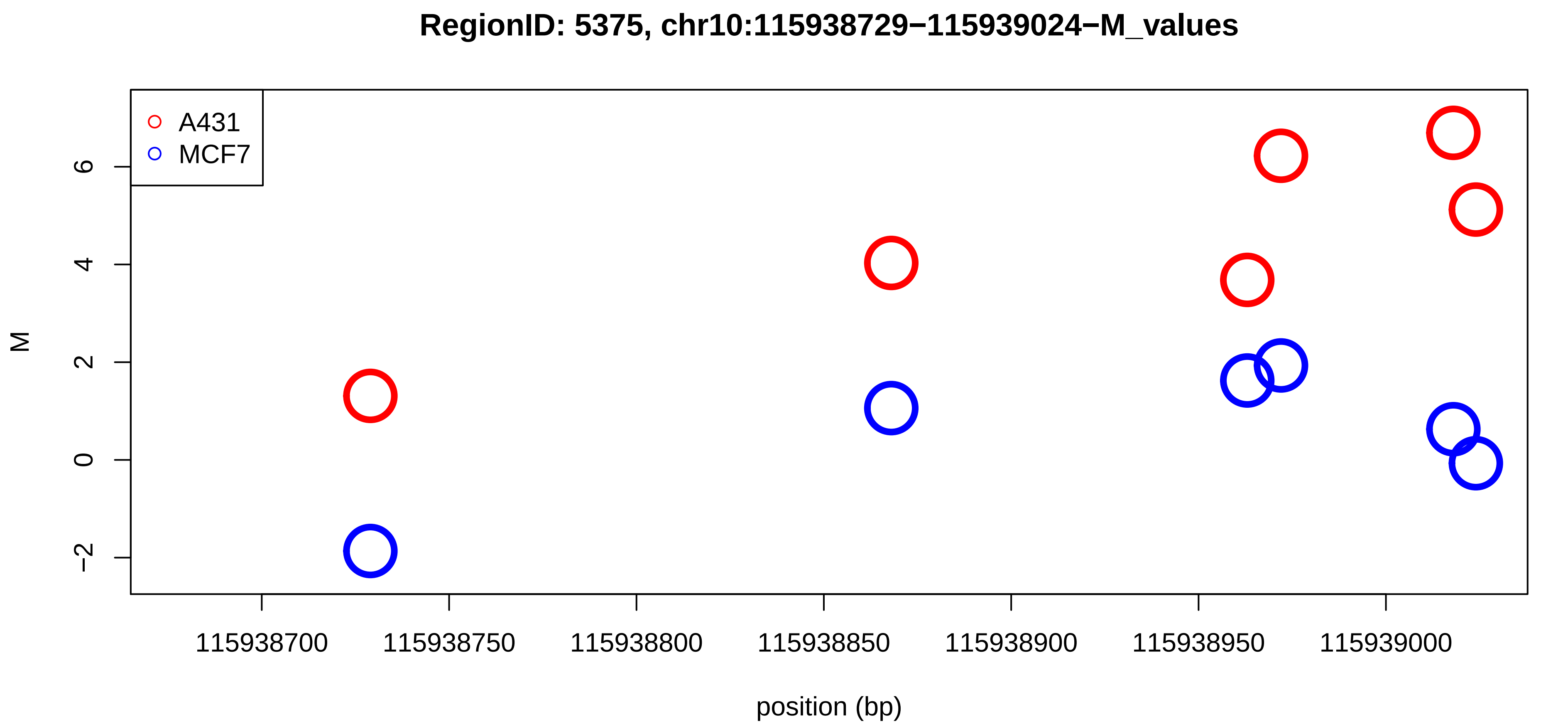
RegionID: 5375, chr10:115938729−115939024−M_values
A431
MCF7
M
position (bp)
115938700
115938750
115938800
115938850
115938900
115938950
115939000
−2
0
2
4
6

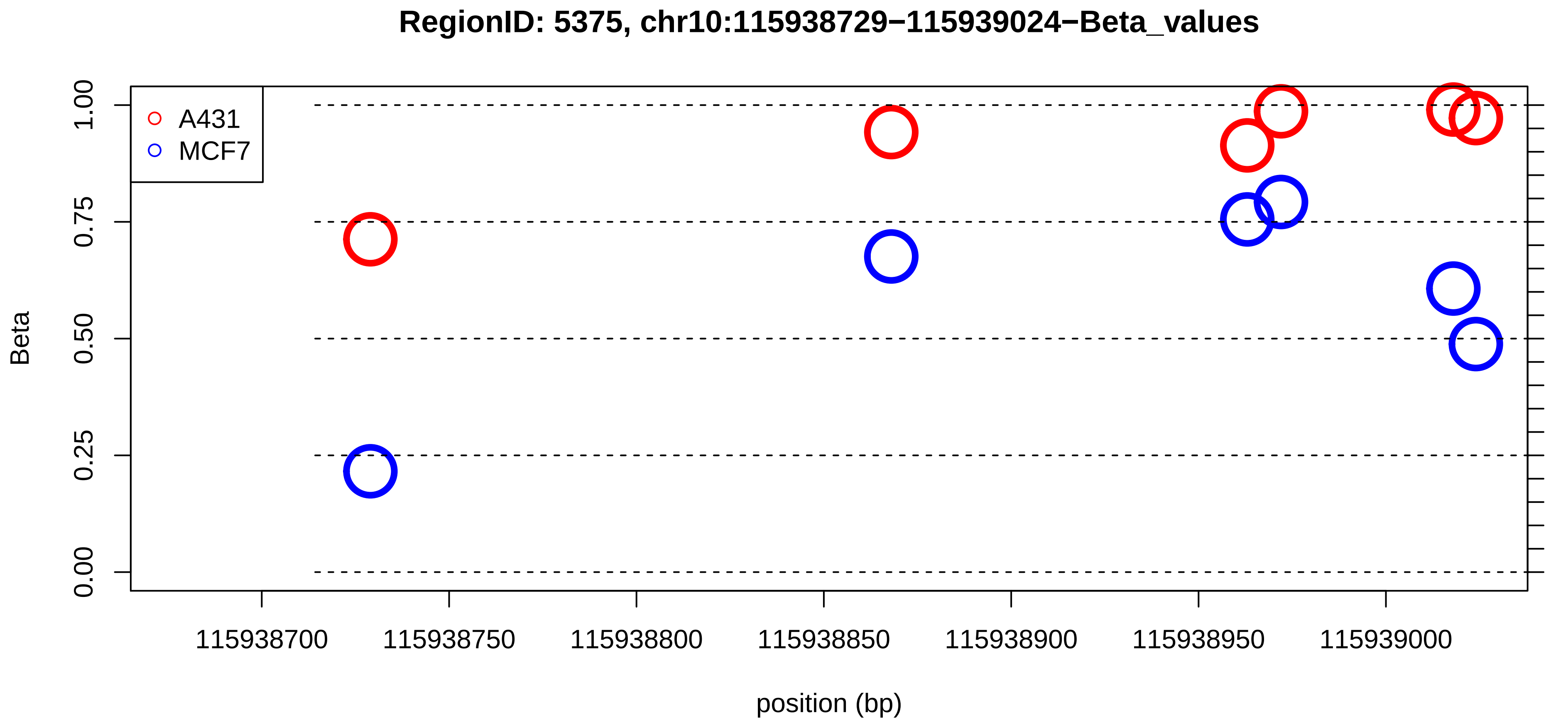
RegionID: 5375, chr10:115938729−115939024−Beta_values
A431
MCF7
Beta
position (bp)
115938700
115938750
115938800
115938850
115938900
115938950
115939000
0.00
0.25
0.50
0.75
1.00

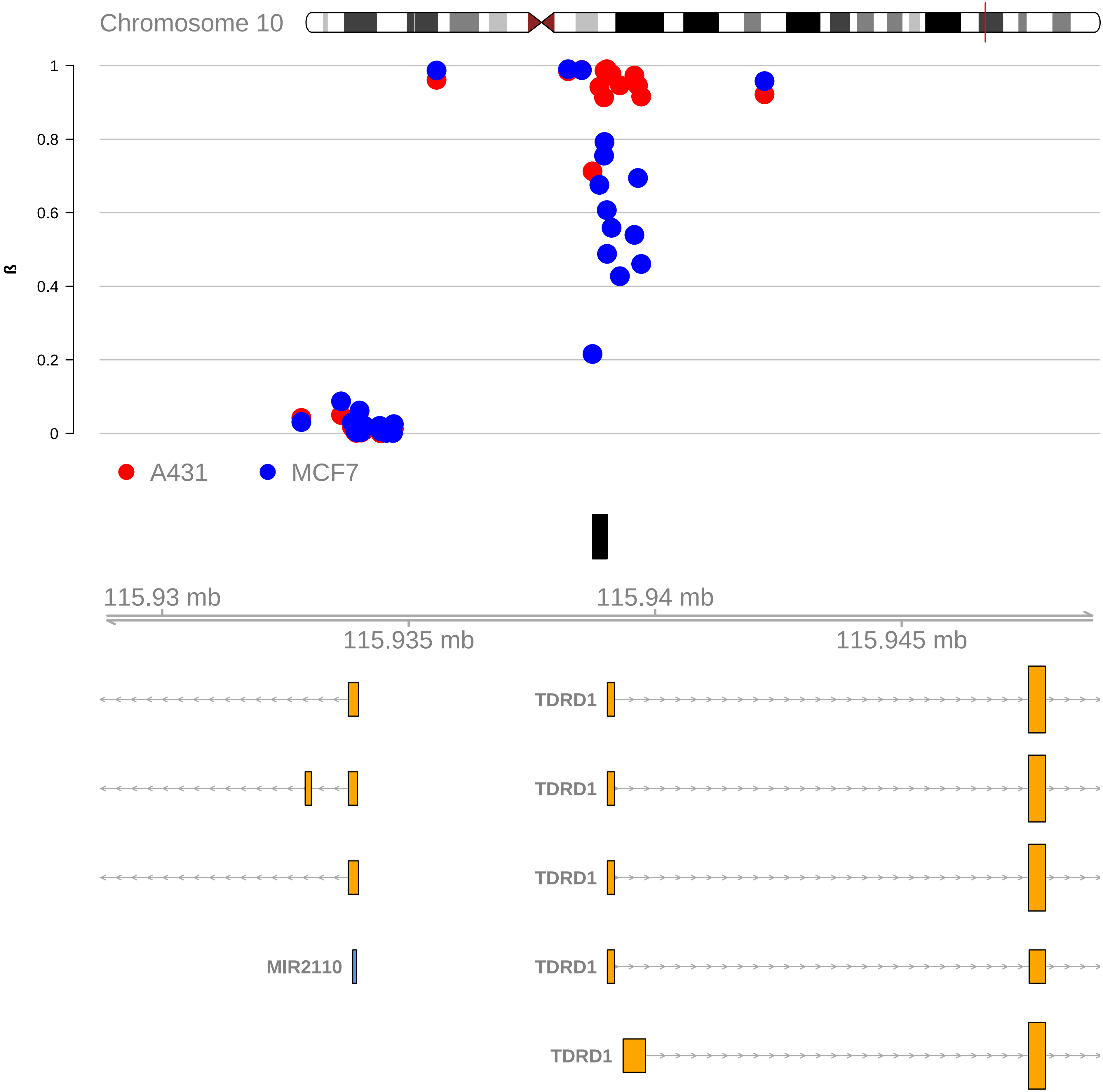

Chromosome 10
1
0.8
0.6
0.4
0.2
0
ß
A431
MCF7
115.93 mb
115.94 mb
115.935 mb
115.945 mb
TDRD1
TDRD1
TDRD1
MIR2110
TDRD1
TDRD1
